# Periodic limb movements in patients with suspected obstructive sleep apnea without comorbid conditions

**DOI:** 10.3389/fmed.2024.1378410

**Published:** 2024-04-26

**Authors:** Christopher Seifen, Moritz Herrmann, Johannes Pordzik, Christoph Matthias, Haralampos Gouveris

**Affiliations:** Sleep Medicine Center & Department of Otolaryngology, Head and Neck Surgery, University Medical Center Mainz, Mainz, Germany

**Keywords:** periodic limb movement, PLM, obstructive sleep apnea, OSA, PLMI, PLMS

## Abstract

**Background:**

Periodic limb movement disorder (PLMD) and obstructive sleep apnea (OSA) are overlapping clinical syndromes with common risk factors. However, current literature has failed to establish a clear pathophysiological link between them. Thus, little is known about periodic limb movements (PLM) in otherwise healthy patients with suspected OSA.

**Methods:**

We performed a retrospective analysis of 112 patients (age: 44.5 ± 12.0 years, 14.3% female) with suspected OSA who underwent full night polysomnography for the first time. Patients with chronic diseases of any kind, recent infections, malignancies, or daily or regular use of any type of medication were excluded. Group comparisons were made based on the severity of OSA (using the apnea hypopnea index, AHI) or the periodic limb movement index (PLMI).

**Results:**

Both, PLMI and the total number of periodic limb movements during sleep (PLMS), showed a significant increase in patients with severe OSA. In addition, AHI and apnea index (AI) were significantly higher in patients with PLMI >15/h, with a similar trend for hypopnea index (HI) (*p* < 0.001, *p* < 0.001, and *p* > 0.05, respectively). PLMI was significantly positive correlated with AHI, AI, and HI (*r* = 0.392, *p* < 0.001; *r* = 0.361, *p* < 0.001; and *r* = 0.212, *p* < 0.05, respectively). Patients with PLMI >15/h were significantly older (*p* < 0.001). There was no significant association between body mass index (BMI) and PLMI >15/h.

**Conclusion:**

We found a significant association between the severity of OSA and PLM in our study population with suspected OSA but without other comorbidities. PLMI and PLMS were significantly increased in patients with severe OSA. Future prospective studies with larger collectives should verify the presented results and should include mechanistic aspects in their evaluation.

## 1 Introduction

Periodic limb movements during sleep (PLMS) are an involuntary sleep-related phenomenon defined as periodic episodes of repetitive limb movements (1). More often than not the lower extremities are affected, with typical dorsiflexion of the toes and ankles and occasional flexion of the hips and knees. In contrast, the upper extremities are less commonly affected (2). Periodic limb movements (PLM) are often described as “Babinski-like” response (3). Scoring criteria require the repetitive movements to last between 0.5 and 5 s (1, 4). To determine the frequency of PLMS occurrence, the periodic limb movement index (PLMI), calculated as the number of PLM per hour of sleep, is commonly used (4). Five PLM per hour of sleep used to be the diagnostic threshold for periodic limb movement disorder (PLMD) (5). However, several studies have shown that healthy individuals without sleep disorders may have more than ten PLM per hour of sleep (6). Therefore, the current guidelines have set the threshold for PLMI in adults at 15 events/h (h) for the diagnosis of PLMD (4). Moreover, the diagnosis of PLMD is a diagnosis of exclusion. Thus, underlying restless legs syndrome (RLS), narcolepsy, or REM sleep behavior disorder (RBD) should be excluded (7). A prevalence of PLMD in 4–11% of the general adult population has been advocated (8).

Community-based studies have highlighted the significance of age, male gender and RLS as independent risk factors for PLMD (9). In addition to RLS, the occurrence of PLMD has been associated with heart disease (10), musculoskeletal disease (10), narcolepsy with cataplexy (10), chronic kidney disease (11), magnesium deficiency (12), neuropsychiatric and neurodegenerative disorders (13), iron deficiency (10), and diabetes mellitus (14). However, the pathophysiological mechanisms underlying these associations are far from clear, particularly with respect to neuropsychiatric and neurodegenerative disorders. Similarly, the use of several psychotropic medications has been linked to PLMD, or PLMI >15/h, for example tricyclic anti-depressants (e.g., amitriptyline), or serotonin and norepinephrine reuptake inhibitors (e.g., venlafaxine and mirtazapine) (15). Lifestyle habits such as increased caffeine consumption, higher body mass index (BMI), and working nights or shifts have also been linked to an increased number of PLM (9, 10).

Although PLM are considered a potential sleep disruptor, their clinical significance is unclear (16). Retrospective studies suggest a high prevalence of comorbid PLM in patients with obstructive sleep apnea (OSA) (16). OSA is known as the most relevant form of sleep-disordered breathing, anatomically defined by partial to complete airway obstruction during sleep, even when respiratory effort is still present (17, 18). The relationship between OSA and PLM is complex, and a precise rationale is unclear. Recently, a large prospective multicenter randomized controlled trial found that guideline-directed therapy for OSA with continuous positive airway pressure (CPAP) had no effect on the severity or expression of PLM (16). These and other studies suggest that the periodicity of OSA and PLM are not caused by a common central generator (16, 19). Moreover, an increasing accumulation of comorbidities has been previously associated with an increased OSA severity, especially in older male subpopulations (20).

To this end, the purpose of this study was to investigate the extent to which PLM vary in otherwise healthy individuals with clinical suspicion of OSA. Through careful patient selection, we aimed to minimize important confounding factors that may independently influence PLM as well as OSA, such as concomitant acute or chronic diseases or daily medication use.

## 2 Methods

### 2.1 Design

In our accredited sleep laboratory, patients receive full-night polysomnography (PSG) for the diagnosis or treatment monitoring of sleep-related breathing disorders. A licensed technician ensures that each PSG is performed correctly, and a board-certified sleep physician accomplishes evaluation (including the assessment of periodic limb movements) according to the American Academy of Sleep Medicine standard guidelines (4).

Our institutional guidelines require an outpatient polygraphy (PG) with a home sleep-apnea testing device as the initial diagnostic approach for patients suspected of having a sleep-related breathing disorder. An additional PSG is performed to validate an accurate diagnosis, or to definitively exclude a sleep-related breathing disorder when results are inconclusive. Ultimately, PSG is performed to initiate the optimal therapeutic management of a sleep-related breathing disorder.

For this study, we screened our clinical database from January 1, 2020, to December 31, 2022, for all patients who underwent PSG for the first time. In an initial step, all patients with a technically qualitative PSG were included in the study, provided that a diagnosis of OSA was made or a clinically relevant sleep-related breathing disorder was ruled out. The diagnosis of any type of sleep-related breathing disorder other than OSA (e.g., periodic breathing or Cheyne-Stokes breathing) resulted in study exclusion.

Then, the medical records of these patients were searched to obtain information on age, BMI, and sex. Likewise, the medical records were screened for information on chronic diseases of any types (e.g., type 2 diabetes, arterial hypertension, pulmonary disease, cardiovascular disease, chronic mental disorders, or any others, e.g., RLS), recent infections, malignancies, and daily or regular (e.g., weekly, monthly) use of medications of any types. In this next step, only patients who were at least 18 years of age and did not have any recorded comorbidities, namely any chronic condition, recent infections, or malignancies, and were not taking medications of any types were further considered for study inclusion.

In all included patients, each PSG recording was analyzed for selected parameters (in alphabetical order):

- AHI: apnea hypopnea index: apneas and hypopneas/h;
- AI: apnea index: apneic events/h;
- ARI: arousal index: number of arousals/h of sleep;
- HI: hypopnea index: hypopnea events/h;
- ODI: oxygen desaturation index: average number of desaturation episodes (decrease in the mean oxygen saturation of ≥3%) per hour;
- PLMI: periodic limb movement index: total number of periodic limb movements per hour of sleep;
- PLMS: periodic limb movements during sleep (total number);
- T90: percentage of cumulative time with peripheral oxygen saturation below 90% in total sleep time; and
- TST: total sleep time in minutes.

For further analysis, groups were formed based on OSA severity on the one hand or the presence of a pathological PLMI on the other. Based on epidemiological studies, the PLMI cut-off has been set to >15/h in adults when a diagnosis of PLMD is considered (4). However, PLMS can be scored according to the criteria recommended by the (above mentioned) AASM or the criteria recommended by the World Association of Sleep Medicine (WASM) (4, 21). In general, limb movements are more frequent after respiratory events than before and during respiratory events, and more respiratory-related leg movements (RRLM) may be scored based on WASM criteria than based on AASM criteria (22).

Grouping based on OSA severity (4):

- all male and female patients with an AHI of <5/h (“none”);
- all male and female patients with an AHI of ≥5 to <15/h (“mild”);
- all male and female patients with an AHI of 15 to 30/h (“moderate”); and
- all male and female patients with an AHI of >30/h (“severe”).

Grouping based on PLMI (4):

- all male and female patients with an PLMI of <15/h (“PLMI <15/h”); and
- all male and female patients with an PLMI of >15/h (“PLMI >15/h”).

**Figure 1:**
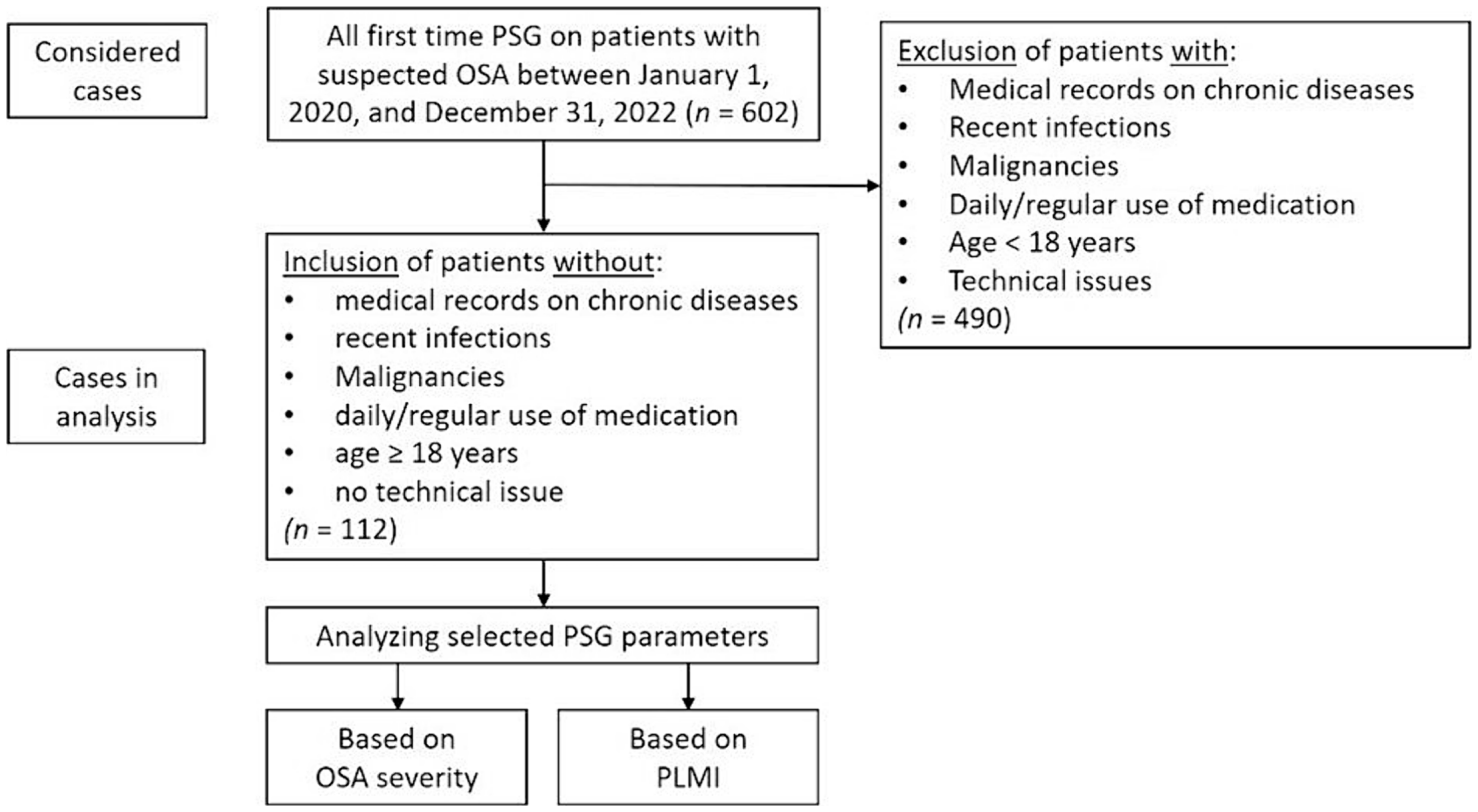
Flowchart for patient selection and study design. OSA, obstructive sleep apnea; PLMI, periodic limb movement index; PSG, polysomnography.

For a better understanding of the patient selection and study design, see the flowchart in Figure 1.

### 2.2 Statistics

GraphPad Prism Version 5.01 (GraphPad Software, Boston, MA, United States) was used for statistical analysis and graphical representation. Normally distributed values were described with mean and standard deviation (SD). Non-normally distributed values were described with median and interquartile range (IQR). The Kolmogorov–Smirnov test was used to analyze whether the values originated from a Gaussian distribution. If the values were normally distributed, comparisons between three groups (e.g., “no/mild OSA” vs. “moderate OSA” vs. “severe OSA”) were performed using one-way ANOVA with Tukey’s *post hoc* test. If no Gaussian distribution was found, then the comparison between three groups was carried out using the Kruskal-Wallis test and the Dunn’s *post hoc* test. The comparison between the two groups of PLMD-severity (e.g., “PLMI <15/h” vs. “PLMI >15/h”) was performed with an unpaired *t*-test if the distribution of values was normal, or with a Mann–Whitney test if no Gaussian distribution could be assumed. The Chi-square test or Fisher’s exact test were used to test whether the proportion of female patients differed significantly between the groups. To test the correlation, Spearman’s correlation coefficient was calculated. We considered the results significant if the *p*-value was <0.05 (*), *p* < 0.01 (**) and *p* < 0.001 (***). Boxplots representing median and IQR were used in the figures.

### 2.3 Ethics

In this study, only health data that is routinely collected in the clinic was analyzed retrospectively. So-called “third parties” did not have access to the data. The ethics committee of the State Medical Association of Rhineland-Palatinate approved the protocol (2023-17,345).

## 3 Results

### 3.1 Study population characteristics

Between January 1, 2020, to December 31, 2022, *n* = 602 patients underwent full night PSG for the first time in our accredited sleep laboratory. After considering all inclusion and exclusion criteria, *n* = 112 patients were included in the present study, 96 (85.7%) male and 16 (14.3%) female. The mean age of the study population was 44.5 ± 12.0 years, and the median BMI was 28.0 (25.0–30.0) kg/m^2^.

After categorizing the study population based on AHI, one patient was included in the “none” OSA group (AHI < 5/h), 35 patients were included in the “mild” OSA-severity group (AHI ≥ 5/h but <15/h), 42 patients were included in the “moderate” OSA-severity group (AHI 15–30/h) and 34 patients were included in the “severe” group (AHI > 30/h). To create more suitable group structures for the subsequent statistical analysis, the “none” and “mild” OSA-severity groups were combined into a new “none/mild” OSA-severity group. This measure was taken due to the only patient with an AHI of <5/h, which made a statistical analysis of such subgroup impracticable.

### 3.2 Association of periodic limb movements with obstructive sleep apnea

**Figure 2:**
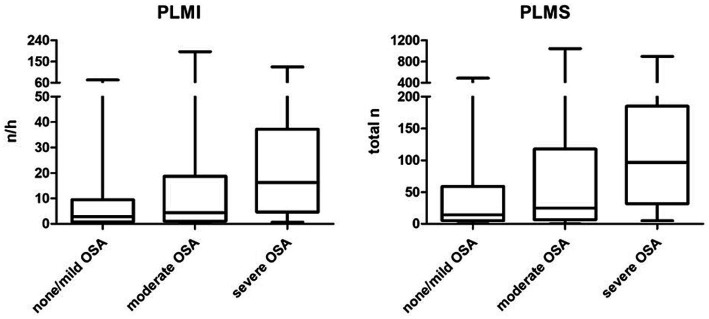
Periodic limb movements based on the severity of obstructive sleep apnea. OSA, obstructive sleep apnea; PLMI, periodic limb movement index; PLMS, periodic limb movements during sleep.

In a first step, the PLM (divided into PLMI and PLMS) were assessed according to the severity of OSA using the AHI. PLMI and PLMS showed a significant increase in patients with severe OSA, while both metrics did not differ significantly between patients with no or mild OSA and those with moderate OSA. T90 and ODI, both important metrics for the additional objective assessment of the severity of OSA, also increased significantly with AHI. Another finding was that a total of *n* = 38 patients (33.9% of the study population) had a PLMI >15/h. The proportion of female patients decreased with increasing severity of OSA, but without statistical significance. Detailed information about statistics and graphical representations of the PLMI and PLMS, as well as the epidemiological data and polysomnographic parameters of the study groups, can be found in Table 1 and Figure 2.

**Table 1:** Comparison of periodic limb movements during sleep and periodic limb movement index among the three patient groups based on the severity of obstructive sleep apnea.

	None/mild	Moderate	Severe	Between group comparison (*p*-value)
Number of patients	36	42	34	
Number of female patients (%)	8 (22.2)	7 (16.7)	1 (2.9)	Not significant
Age in years (± SD)	41.5 ± 13.0	44.2 ± 12.8	47.8 ± 9.2	Not significant
BMI in kg/m^2^ (IQR)	26.2 (24.0–28.0)	28.0 (25.6–30.0)	29.5 (27.0–33.3)	< 0.05 for none/mild vs. moderate, < 0.001 for none/mild vs. severe
AHI in n/h (± SD)	9.3 ± 3.1	22.6 ± 4.3	53.2 ± 19.2	< 0.001 for none/mild vs. moderate, < 0.001 for none/mild vs. severe, < 0.001 for moderate vs. severe
ODI in n/h (± SD)	8.2 ± 4.6	19.2 ± 6.5	50.1 ± 21.7	< 0.001 for none/mild vs. moderate, < 0.001 for none/mild vs. severe, < 0.001 for moderate vs. severe
T90 in % (IQR)	0.1 (0.0–0.4)	0.6 (0.1–1.5)	3.8 (1.1–13.4)	< 0.01 for none/mild vs. moderate, < 0.001 for none/mild vs. severe, < 0.001 for moderate vs. severe
PLMI in n/h (IQR)	2.8 (0.8–9.5)	4.3 (1.0–18.7)	16.3 (4.7–37.2)	< 0.001 for none/mild vs. severe, < 0.01 for moderate vs. severe
PLMS in *n* (IQR)	14.0 (5.0–59.0)	25.0 (6.5–117.8)	96.5 (31.5–185.0)	< 0.001 for none/mild vs. severe, < 0.01 for moderate vs. severe
TST in min (± SD)	361.2 ± 58.2	374.1 ± 51.0	378.0 ± 51.4	Not significant
ARI in n/h (± SD)	12.7 ± 7.0	15.1 ± 7.0	23.9 ± 12.7	< 0.001 for none/mild vs. severe, < 0.001 for moderate vs. severe

“None/mild”-all male and female patients with apnea hypopnea index (AHI) < 15/h; “moderate”-all male and female patients with AHI 15–30/h; “severe”-all male and female patients with AHI > 30/h. AHI, apnea hypopnea index; ARI, arousal index; BMI, body-mass index; IQR, interquartile range; ODI, oxygen desaturation index; PLMI, periodic limb movement index; PLMS, periodic limb movements during sleep; SD, standard deviation; T90, percentage of cumulative time with oxygen saturation below 90% in total sleep time; TST, total sleep time.

**Figure 3:**
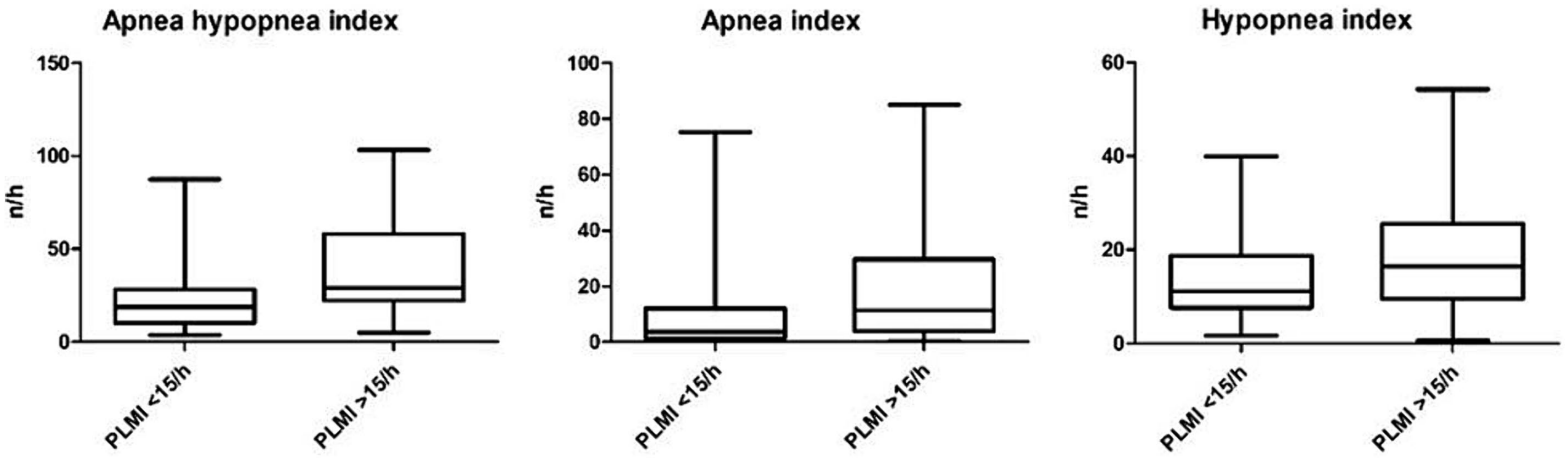
Apnea hypopnea index, apnea index, and hypopnea index based on the periodic limb movement index.

In a next step, different polysomnographic parameters were analyzed and compared based on the PLMI. AHI and AI were significantly elevated in group “PLMI >15/h.” Accordingly, HI showed a clear tendency of higher values according to elevated PLMI but slightly without statistical significance. The proportion of female patients was lower in group “PLMI <15/h” compared to group “PLMI >15/h, but without statistical significance. The aforementioned and other polysomnographic parameters are detailed on Table 2 and shown in Figure 3.

**Table 2:** Comparison of polysomnographic parameters between the two patient groups based on the periodic limb movement index.

	PLMI < 15/h	PLMI > 15/h	Between group comparison (*p*-value)
Number of patients	74	38	
Number of female patients (%)	12 (16.2)	4 (10.5)	Not significant
Age in years (± SD)	41.3 ± 11.8	50.6 ± 10.1	< 0.001
BMI in kg/m^2^ (IQR)	27.5 (25.0–30.0)	28.0 (24.0–32.0)	Not significant
PLMI in n/h (IQR)	2.2 (0.8–5.3)	29.3 (19.4–40.4)	< 0.001
PLMS in *n* (IQR)	12.5 (5.0–35.0)	158.0 (129.8–281.5)	< 0.001
AHI in n/h (IQR)	18.8 (10.2–28.5)	29.1 (22.3–58.2)	< 0.001
AI in n/h (IQR)	3.7 (1.4–12.1)	11.4 (4.0–29.7)	< 0.001
HI in n/h (IQR)	11.5 (7.7–18.7)	16.5 (9.6–25.5)	Not significant
ODI in n/h (IQR)	15.5 (8.6–25.0)	26.3 (17.3–58.9)	< 0.001
T90 in % (IQR)	0.4 (0.0–2.1)	1.0 (0.3–13.1)	< 0.01
TST in min (± SD)	372.5 ± 49.4	368.4 ± 61.4	Not significant
ARI in n/h (IQR)	12.9 (8.5–18.9)	18.3 (13.0–29.1)	< 0.01

“PLMI < 15/h”-all male and female patients with periodic limb movement index < 15/h; “PLMI > 15/h”-all male and female patients with periodic limb movement index > 15/h. AHI, apnea hypopnea index; AI, apnea index; ARI, arousal index; BMI, body-mass index; IQR, interquartile range; HI, hypopnea index; ODI, oxygen desaturation index; PLMI, periodic limb movement index; PLMS, periodic limb movements during sleep; SD, standard deviation; T90, percentage of cumulative time with oxygen saturation below 90% in total sleep time; TST, total sleep time.

### 3.3 Different correlations with the periodic limb movement index

**Figure 4:**
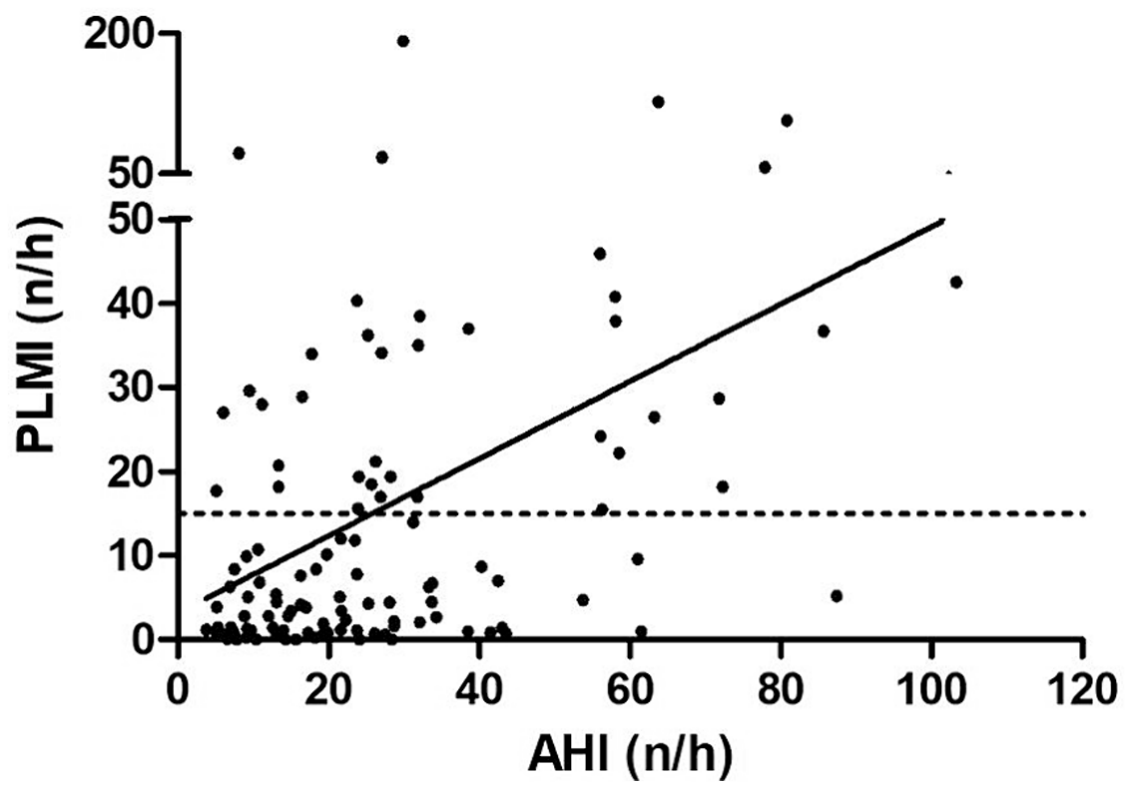
Correlation between apnea hypopnea index and periodic limb movement index. The dashed line shows the cut-off value at a periodic limb movement index of 15/h and the solid line shows the correlation. AHI, apnea hypopnea index; PLMI, periodic limb movement index.

Finally, correlation analyses were performed to ascertain possible factors associated with the existence and severity of PLMS. Spearman’s correlation analysis identified a significant positive correlation between PLMI and age, AHI, AI, HI, ODI and T90 (*p* < 0.05, *p* < 0.001, *p* < 0.001, *p* < 0.05, *p* < 0.001 and *p* < 0.05, respectively). Contrarily, PLMI and BMI were not significantly correlated (*p* > 0.05). The correlation analyses are presented on Table 3. The example correlation between PLMI and AHI is shown in Figure 4.

**Table 3:** Different correlations with the periodic limb movement index.

*x*	*y*	*r*	*p*-value
Age	PLMI	0.239	< 0.05
BMI	PLMI	0.035	> 0.05
AHI	PLMI	0.392	< 0.001
AI	PLMI	0.361	< 0.001
HI	PLMI	0.212	< 0.05
ODI	PLMI	0.397	< 0.001
T90	PLMI	0.223	< 0.05

AHI, apnea hypopnea index; AI, apnea index; BMI, body-mass index; HI, hypopnea index; ODI, oxygen desaturation index; PLMI, periodic limb movement index; T90, percentage of cumulative time with oxygen saturation below 90% in total sleep time.

## 4 Discussion

Studies addressing the impact of underlying OSA on PLM in patients with no comorbidities have been lacking. The present study therefore aimed to investigate the extent to which OSA and PLM are related or the severity of OSA influences PLM. For this purpose, in this study only (suspected) OSA patients without any other known comorbidities were included.

We provide evidence that PLM, divided into PLMI and PLMS, increased with OSA severity in OSA patients without any comorbid conditions. However, PLMI and PLMS did not differ significantly between patients with no or mild OSA and those with moderate OSA. In addition, AHI and AI were significantly higher in patients with PLMI >15/h, with a similar (although not reaching statistical significance) trend for HI. A significant positive correlation was found between PLMI and AHI, AI, and HI. In our study population, age was significantly higher in patients with a PLMI >15/h, whereas BMI tended to be similar in patients with relevant PLM and those without. In addition, the proportion of female patients was lower in the study subgroup with PLMI >15/h. The prevalence of patients with PLMI >15/h was 33.9% in our study, higher than the previously reported 5–8% in adult community-based cohorts (23, 24). The higher prevalence of PLMI >15/h in our study could be the result of a selection bias, a higher proportion of male patients in this relatively small study population, or environmental factors such as measurement of PLM in a sleep laboratory. In addition, TST was comparable in patients with low and high PLMI, while ARI as a common sleep macrostructure metric was significantly higher in patients with a PLMI >15/h.

PLMD causes sleep fragmentation that may disturb underlying sleep rhythms and their intrinsic functions. Expected, patients affected by PLMD frequently complain about daytime sleepiness, poor concentration, or non-restorative sleep (25). Patients with OSA often report a similar constellation of symptoms. Although PLMD and OSA share overlapping symptoms, community-based studies have failed to detect a direct linkage between these conditions (9, 26). A possible explanation was provided previously in a study in which different periodicities of PLM and OSA before and after treatment with continuous positive airway pressure (CPAP) were found, suggesting that both conditions are not generated by a common central generator (19). Findings from another study that investigated coupling of electroencephalography and either chin- or leg-electromyography signals of PSG in OSA patients support the theory of a distinct central generator between OSA and PLMS (27).

However, another study showed PLM to increase in moderate to severe OSA after CPAP treatment, presumably due to “unmasking” underlying PLMD (28). The literature on the topic of correlation between PLM and severity of OSA, as depicted by the AHI, has been inconsistent. One study found a significant positive correlation between PLM and AHI (29). These findings are in line with the results of the present study. In contrast, another study found evidence for no significant correlation between PLM and AHI (16). It should be noted that the above studies (other than the present study) attempted to minimize, but they did not exclude at last, confounding factors, such as possible influence of medications on PLM or severe comorbid conditions in their study populations.

Interestingly, in the HypnoLaus study, high PLMS was found mainly in middle-aged Europeans; in particular, the authors found age and male gender to be independent predictors of increased PLMS (9). This trend holds true for the results in the present study and is consistent with further previous studies (29). Conversely, increased BMI has been associated with an increased risk of PLMI >15/h (9). However, in the present study, BMI was approximately identical in the group with PLMI <15/h and the group with PLMI >15/h.

The major strength of our study is the strict selection of patients, as we extensively filtered the database of our sleep laboratory for those patients whose medical records excluded chronic diseases of any types, recent infections, malignancies, or daily or regular use of medications of any types. Through these measures, a possible impact of important confounders that have an influence on PLM could be kept to a minimum.

We acknowledge several limitations of the present study. One major limitation is that the results are based on observations in retrospective analyses. Because this study is observational, further randomized controlled and prospective trials are needed to validate the presented findings. Another important limitation is the lack of information on various lifestyle factors such as nicotine and alcohol consumption and night or shift work. In addition, other characteristics of the study population characteristics (e.g., head and neck circumference, Mallampati score, or body habitus other than BMI) were not included in the present study. These factors could have independently impact on the reported PLM. In addition, it cannot be ruled out that any condition (e.g., neurological, or psychiatric) was already present, although clinically unnoticed, at the time of data collection and were therefore not yet diagnosed at that time. This limitation may be better addressed in the future using a longitudinal study design. Also, the reported results refer to predominantly male study participants, especially in the group with severe OSA. We must acknowledge this gender bias as a major limitation that may have independently confounded the reported results. Regarding the female population and because of the retrospective data analysis, it was not possible to determine the premenopausal or postmenopausal status of the included female study participants. Ultimately, it should be considered that the PLM index is subject to high night-to-night variability (30–32), which could independently have limited the significance of these polysomnographic single-night measurements. In addition, the assessment of PLM is particularly challenging in patients with severe OSA, as limb movements associated with periodic obstructive respiratory events (i.e., respiratory-related leg movements; RRLM) can “mimic” PLM (33).

The results of the present study cannot answer the question of a possible common central generator of OSA and PLM. However, the finding of increased PLMS in patients with clinically relevant OSA suggests that there may be a link between these two conditions: if not in the form of a periodicity, then perhaps in the form of mutual reinforcement.

Future prospective studies need to focus on the above limitations and examine them in larger study populations to validate the reported results. In addition, future studies should include additional biomarkers in their evaluation, e.g., as the intracellular storage protein ferritin (34–36).

## 5 Conclusion

Severe OSA is significantly associated with increased and likely clinically relevant PLM in OSA patients without comorbid conditions. A mechanistic link cannot be inferred from this observation and thus should be investigated in future studies. Additionally, prospective studies should verify the presented results.

## Data availability statement

The raw data supporting the conclusions of this article will be made available by the authors, without undue reservation.

## Ethics statement

The studies involving humans were approved by Ethics committee of the State Medical Association of Rhineland-Palatinate. The studies were conducted in accordance with the local legislation and institutional requirements. Written informed consent for participation was not required from the participants or the participants’ legal guardians/next of kin in accordance with the national legislation and institutional requirements.

## Author contributions

CS: Conceptualization, Data curation, Formal analysis, Investigation, Methodology, Visualization, Writing – original draft, Writing – review & editing. MH: Writing – review & editing. JP: Writing – review & editing. CM: Writing – review & editing. HG: Conceptualization, Formal analysis, Supervision, Writing – review & editing.
